# Supplementary material for: Causal Relationship between Aging and Anorexia Nervosa: A White-Matter-Microstructure-Mediated Mendelian Randomization Analysis
Source: Biomedicines. 2024 Aug 16;12(8):1874. doi: 10.3390/biomedicines12081874 (PMC11351342; doi:10.3390/biomedicines12081874)
Supplement: Supplementary file 1 [file biomedicines-12-01874-s001.zip › Supplementary Figure/Figure S4/Singe SNP plot of MR analysis for TL to ubm-b-1746.pdf]

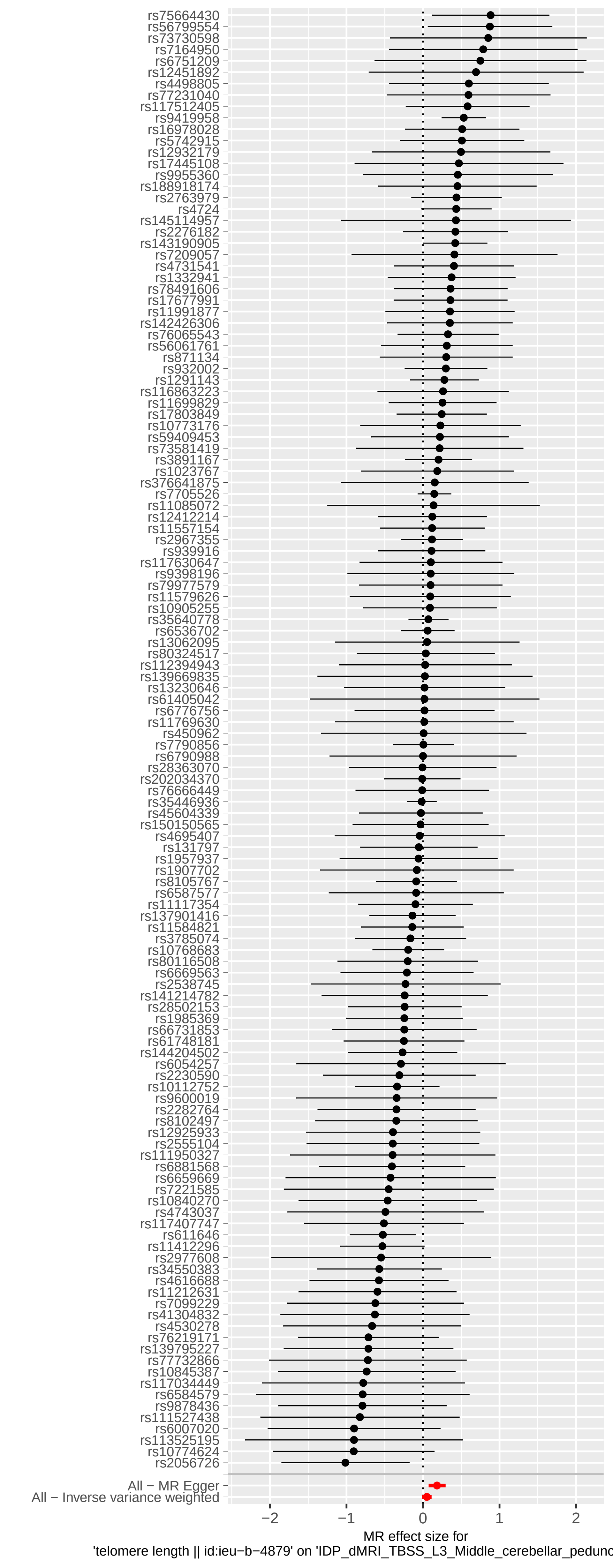
rs75664430
rs56799554
rs73730598
rs7164950
rs6751209
rs12451892
rs4498805
rs77231040
rs117512405
rs9419958
rs16978028
rs5742915
rs12932179
rs17445108
rs9955360
rs188918174
rs2763979
rs4724
rs145114957
rs2276182
rs143190905
rs7209057
rs4731541
rs1332941
rs78491606
rs17677991
rs11991877
rs142426306
rs76065543
rs56061761
rs871134
rs932002
rs1291143
rs116863223
rs11699829
rs17803849
rs10773176
rs59409453
rs73581419
rs3891167
rs1023767
rs376641875
rs7705526
rs11085072
rs12412214
rs11557154
rs2967355
rs939916
rs117630647
rs9398196
rs79977579
rs11579626
rs10905255
rs35640778
rs6536702
rs13062095
rs80324517
rs112394943
rs139669835
rs13230646
rs61405042
rs6776756
rs11769630
rs450962
rs7790856
rs6790988
rs28363070
rs202034370
rs76666449
rs35446936
rs45604339
rs150150565
rs4695407
rs131797
rs1957937
rs1907702
rs8105767
rs6587577
rs11117354
rs137901416
rs11584821
rs3785074
rs10768683
rs80116508
rs6669563
rs2538745
rs141214782
rs28502153
rs1985369
rs66731853
rs61748181
rs144204502
rs6054257
rs2230590
rs10112752
rs9600019
rs2282764
rs8102497
rs12925933
rs2555104
rs111950327
rs6881568
rs6659669
rs7221585
rs10840270
rs4743037
rs117407747
rs611646
rs11412296
rs2977608
rs34550383
rs4616688
rs11212631
rs7099229
rs41304832
rs4530278
rs76219171
rs139795227
rs77732866
rs10845387
rs117034449
rs6584579
rs9878436
rs111527438
rs6007020
rs113525195
rs10774624
rs2056726
All − MR Egger
All − Inverse variance weighted
−2
−1
0
1
2
MR effect size for
'telomere length || id:ieu−b−4879' on 'IDP_dMRI_TBSS_L3_Middle_cerebellar_pedunc
